# Assessment of Convolutional Neural Network Pre-Trained Models for Detection and Orientation of Cracks

**DOI:** 10.3390/ma16020826

**Published:** 2023-01-14

**Authors:** Waqas Qayyum, Rana Ehtisham, Alireza Bahrami, Charles Camp, Junaid Mir, Afaq Ahmad

**Affiliations:** 1 Department of Civil Engineering, University of Engineering and Technology, Taxila, Rawalpindi 46600, Pakistan; 2 Department of Building Engineering, Energy Systems, and Sustainability Science, Faculty of Engineering and Sustainable Development, University of Gävle, 801 76 Gävle, Sweden; 3 Department of Civil Engineering, University of Memphis, Memphis, TN 38152, USA; 4 Department of Electrical Engineering, University of Engineering and Technology, Taxila, Rawalpindi 46600, Pakistan

**Keywords:** convolutional neural networks, orientation of cracks, cracks detection, deep learning, pre-trained models

## Abstract

Failure due to cracks is a major structural safety issue for engineering constructions. Human examination is the most common method for detecting crack failure, although it is subjective and time-consuming. Inspection of civil engineering structures must include crack detection and categorization as a key component of the process. Images can automatically be classified using convolutional neural networks (CNNs), a subtype of deep learning (DL). For image categorization, a variety of pre-trained CNN architectures are available. This study assesses seven pre-trained neural networks, including GoogLeNet, MobileNet-V2, Inception-V3, ResNet18, ResNet50, ResNet101, and ShuffleNet, for crack detection and categorization. Images are classified as diagonal crack (DC), horizontal crack (HC), uncracked (UC), and vertical crack (VC). Each architecture is trained with 32,000 images equally divided among each class. A total of 100 images from each category are used to test the trained models, and the results are compared. Inception-V3 outperforms all the other models with accuracies of 96%, 94%, 92%, and 96% for DC, HC, UC, and VC classifications, respectively. ResNet101 has the longest training time at 171 min, while ResNet18 has the lowest at 32 min. This research allows the best CNN architecture for automatic detection and orientation of cracks to be selected, based on the accuracy and time taken for the training of the model.

## 1. Introduction

A significant amount of money is spent each year to identify flaws in infrastructures, primarily buildings, roads, and bridges [1,2]. These infrastructures are constantly under stress due to natural and man-made hazards, such as earthquakes, blasts, and daily use. These stresses cause a variety of deteriorations, one of which is crack development [3,4]. Automation detection of these defects can significantly reduce the time and cost associated with inspection. A building’s occupants and structural integrity are better protected by detecting and analyzing structural flaws before a significant earthquake. Structural cracks may be detected early and remedied more effectively using low-cost monitoring that delivers an early danger alert. Deep learning (DL) is a viable option for supervised or unsupervised feature extraction and transformation, as well as pattern analysis and classification. DL makes use of several layers of non-linear information processing [5]. For instance, an image comprises an array of pixel values. The output from the first layer represents the presence of the edges in the image at a specific direction and location within the image. The second layer can tell the pattern apart thanks to its ability to recognize how the edges are arranged, even if there are small changes in where the edges are. In the third layer, different patterns combine to form a larger group that turns into an identifiable component. Then, in the next layer, the item is recognized based on the combination of these components [6].

A convolutional neural network (CNN) is a branch of DL that processes images to prioritize unique picture attributes to differentiate between images. Initially, CNNs were only used to solve fundamental issues, such as handwritten digit identification; however, CNN-based techniques have become the industry standard for image classification, object location, and picture segmentation [7].

CNN pre-trained architectures have been utilized by researchers for image identification and object detection. Krizhevsky et al. [8] trained a deep CNN named Alexnet to classify 1.2 million images using the ImageNet dataset into 1000 classes. He et al. [9] developed a residual learning framework (ResNet) that was trained with the ImageNet dataset. Performance of ResNet was awarded first prize on the ImageNet Large Scale Visual Recognition Challenge (ILSVRC) 2015 classification task. Howard et al. [10] developed models for mobile and embedded vision applications called MobileNets. The performance of MobileNet was evaluated and compared with other popular models on ImageNet classification. It was highly effective across many applications, such as object identification, fine-grain classification, face characteristics, and large-scale geolocalization. GoogLeNet model was proposed in ILSVRC 14, the quality of the architecture was assessed based on classification and detection [11]. There are many other pre-trained neural networks, such as Inception-V3 [12], VGG16 and VGG19 [13,14], DenseNet [15], ResNet [16], Inception-ResNet [17], DarkNet [18], Xception [18], EfficientNet [19], ShuffleNet [20], and SqueezeNet [21].

The basic CNN architecture consists of convolution, pooling, fully connected, and non-linearity layers. Several non-linear operations are the most commonly used: sigmoid, tanh, and ReLU. Most of the previous research has been carried out on classifying cracked and uncracked images. A little work has been conducted on classifying the images based on the orientation. The orientation of cracks is important as it suggests causes of failures.

This study applies seven well-known pre-trained CNN models and compares their performance in detecting cracks. Each model’s training duration is recorded, and models are evaluated using test images. In the end, the best model is suggested after comparing model accuracy and training time. The following section summarizes previous research works on crack detection, including image recognition and DL. Section 3 includes an experimental study that explains the proposed methodology, data acquisition, training and testing dataset, and pre-trained models. Section 4 discusses the results in detail, and the conclusions of the study are reported in Section 5.

## 2. Literature Review

The safety and durability of structures are directly related to the ability to identify cracks quickly and accurately [22]. Several variables affect the outcomes of manual crack detection methods. The findings of such a manual examination are subjective and depend on the inspector’s skill set, and such examinations are performed by analyzing cracks, i.e., their location and widths [23]. In the case of critical infrastructures, a manual inspection might lead to inaccurate damage assessments [24,25]. Because of this, there is a pressing need for automated procedures to overcome manual methods’ limits in detecting fractures in civil engineering infrastructures.

Visual inspection is a traditional monitoring method for crack detection. Inspectors must travel to inspect the bridges and other engineering structures, which can be tedious. Therefore, automation can make the process smooth, economical, and timesaving. Abdel-Qader et al. [26] used four techniques to detect the cracks, i.e., Fast Fourier Transform, Fast Haar Transform, Sobel, and Canny. They found that the Fast Fourier Transform outperformed the other methods. Prasanna et al. [27] utilized the support vector machine (SVM) algorithm for crack detection with a linear kernel function. The effectiveness of this classification method was evaluated using 118 images subdivided into two categories, one containing cracked images and the other with uncracked images. They found an accuracy of 76%. Maniat et al. [28] used Google Street View images to test and train a VGG16 CNN model with an accuracy of 98.6%.

Vu and Duc [29] trained the Inception-V3, VGG16, and ResNet classifiers using an epoch value of 50 and a batch size of 16 for crack detection. For a dataset of 4000 images, the Inception-V3 and VGG16 models had a high accuracy of 99.9%, and ResNet had an accuracy of 97.5%. Cha et al. [30] utilized a vision-based approach to detect concrete cracks using a DL method. They took 40,000 images to train a CNN with a 98.22% accuracy. Chaiyasarn et al. [31] employed a combined CNN and SVM for extracting crack features from RGB images. They utilized the SVM as an alternative to the SoftMax layer to enhance the classification ability and found an accuracy of approximately 86%. Abdel-Qader et al. [32] applied an algorithm based on the principal component analysis (PCA) to find fractures in a concrete bridge. They employed three different PCA approaches: PCA with raw data, a linear structure modeling implemented before PCA with global information, and local information instead of global information. They found an improvement in the local detection with linear modeling compared with global detection. Wang et al. [33] utilized the three AlexNet models, compared them with ChaNet to detect concrete cracks, and found the ChaNet more reliable with an accuracy of 87.91%. Cha and Choi [34] obtained a 98% accuracy when they applied a CNN architecture to predict cracks using a data set of 40,000 images for training and validation. For the identification of fractures in hot-mix asphalt (HMA) and Portland cement concrete (PCC) surfaces, Gopalakrishnan et al. [35] used a single-layer neural net with the Adam algorithm on ImageNet pre-trained VGG16 DCNN features. Ehtisham et al. [36] took the four pre-trained models for the crack detection and orientation for four classes and found the ResNet50 model had an 86.22% accuracy. Ahmed et al. [37] employed the ResNet50 CNN model for pavement cracks detection at the University of Engineering and Technology (UET), Taxila, with a 99.8% accuracy and 100% precision.

Munawar et al. [38] proposed an architecture that utilizes a cycle generative adversarial network in conjunction with 16 convolutional layers (CycleGAN). For their study, images showing cracks in mid- to high-rise structures (five floors or more) in Sydney, Australia, from the year 2000 were obtained employing UAVs and open-source images. Conventionally, just the last convolution layer is used in a CNN network. However, in this network, more than one layer was applied. Guided filtering (GF) and conditional random fields (CRFs) are critical components of the proposed CNN architecture, since they help refine the predicted outputs and produce accurate results. The suggested architecture was tested using damage data from Sydney-based structures (600 images). They found that the suggested deep hierarchical CNN architecture outperformed the other approaches, GF, Baseline (BN), Deep-Crack GF, and SegNet, with a global accuracy of 99.9%. Additionally, the class average accuracy, the mean intersection of overall union classes (IoU), precision, recall, and F1 score were 93.9%, 87.9%, 83.8%, 87.9%, and 85.8%, respectively.

Özgenel and Sorguç [39] analyzed the performance of pre-trained networks, including AlexNet, VGG16, VGG19, GoogLeNet, ResNet50, ResNet101, and ResNet152. They considered the size of the training image database, the depth of the networks, the number of training epochs, and the expandability to other building material types. Guzman-Torres et al. [40] used a DL algorithm to detect and classify micro and macro cracks images of the concrete by selecting custom network architectures. After fine tuning, they investigated the performance of different architectures, network depth, tuning techniques, and transfer learning methods. The performance of the VGG16 model was improved with an accuracy of 99.5% and F1 score of 100%. Qayyum et al. [41] classified cracked and uncracked images of concrete and cracks in the diagonal, horizontal, and vertical directions using GoogLeNet, MobileNet-V2, and Inception-V3. They found that Inception-V3 performed better than the other two networks, with an accuracy of 97.2% for cracked and uncracked images and 92%, 95%, and 96% for diagonal, horizontal, and vertical images, respectively.

Machine learning (ML) may be used to solve various structural engineering problems. Thai [42] reviewed ML application for: (1) structural analysis and design; (2) structural health monitoring and damage detection; (3) structural fire resistance; (4) structural member resistance to various actions; and (5) concrete mechanical properties and mix design. Thai’s objective was to assist the non-ML structural engineering community in developing ML models for practical applications by providing an overview of ML algorithms and basic concepts, codes, ML libraries, and compiled datasets. Mishra et al. [43] discussed the application of Internet of Things technology and the utilization of sensors for structure monitoring. Their objective was to identify various factors that may influence the long-term and short-term integrity of a structure. Additionally, they presented several case studies on actual structures and laboratory testing for monitoring the structural health of civil engineering structures. Nunez et al. [44] simplified the application of ML in concrete technology by surveying and analyzing algorithms employed to calculate the compressive strength of concrete mixes.

Jiang et al. [45] improved the objection detection method by using depth-wise differentiable convolution, an inverse residual network, and a linear bottleneck structure. They tested these models with 5000 images of damaged concrete, such as cracks, spots, exposed rebar, and spalling damage. The inference speed increased by 24.1% for the YOLO-V3 algorithm and 53.5% for the single shot detection (SSD) identification algorithm. The accuracies of the upgraded YOLO-V3 and SSD algorithms were 64.81% and 64.12%, respectively, which were 3.25% and 4.04% better than the original versions. Dung et al. [46] used the VGG16 for the crack detection in gusset plate welded joints of steel bridges with a dataset of 337 images having 64 × 64 pixels and achieved an accuracy of up to 98%. Liu et al. [47] utilized the U-Net- and DCNN-based methods for detecting cracks in concrete with 512 × 512 × 3 features and found the U-Net to be more refined than the DCNN method with high effectiveness, robustness, and accuracy. Ali et al. [48] applied the CNN architecture for structural crack detection and segmentation. The encoder and decoder architectures, such as SegNet, U-Net, and FCN are the more elegant for fine crack segmentation. Asadi Shamsabadi et al. [49] developed a vision transformer (ViT)-based technique for detecting cracks in concrete and asphalt surfaces applying DeepLabV3+ and U-Net of CNN models.

The use of DL is not limited to the detection of cracks in concrete. Yin et al. [50] employed a CNN-based object detection algorithm named YOLO-V3 to detect defects in drainage systems, including breaks, holes, deposits, cracks, fractures, and roots, and a single type of construction feature—tap. The model was trained with a 4056-image dataset. The framework’s performance had a mean average precision of 85.37%. The research led to labeled closed-circuit television (CCTV) videos, where each frame showed the type of defect and information about it. Hassan et al. [51] proposed a defect classification system using CNN. The primary purpose was to combine CCTV video with the classification system. A dataset of 4702 images for six defects, including longitudinal defects, debris silty, joint faulty, joint open, lateral, and surface damage, was extracted from the CCTV videos. The highest recorded accuracy was 96.33%.

Masonry structures are more earthquake-prone than non-masonry structures. In developing countries, masonry structures are not reinforced to withstand the lateral force produced by seismic loads. About 77% of the estimated damage is due to the collapse of masonry structures. Wang et al. [52] proposed a DL technique to classify confined and unconfined buildings. The dataset consisted of street view images gathered from streets in the Oaxaca State, Mexico, using a 360-degree camera mounted on vehicles. The model could be utilized on a large scale to find the buildings that need retrofitting. Kim et al. [53] proposed a method for fine-tuning LeNet-5 with the METU dataset, which led to the formation of OLeNet. The performance of the newly built model, VGG16, Inception, and ResNet, was evaluated after training the model with 40,000 cracked and uncracked images. The proposed model produced a validation accuracy of 99.8% with an epoch value of just 19. The training time was as low as 220 s. Nguyen et al. [54] developed a model consisting of two stages based on CNN architectures. The aim was to combine the detection and segmentation of road crack images at a pixel level in a single framework. A double-stage framework works significantly well on low-quality, noisy images, and imbalanced datasets. The F1 score for the model was more than 90%. Ali et al. [55] proposed CNN models that were customized for crack detection in concrete structures. The model’s performance was compared with VGG16, VGG10, ResNet50, and Inception-V3 based on precision, computational time, accuracy, results of crack localization, F1 score, and recall. VGG16 and the proposed model performed better compared with other selected architectures. Different methods based on image processing are adopted to detect cracks in a structure. Table 1 lists a summary of these papers.

## 3. Experimental Study

### 3.1. Overview of Methodology

**Figure 1:**
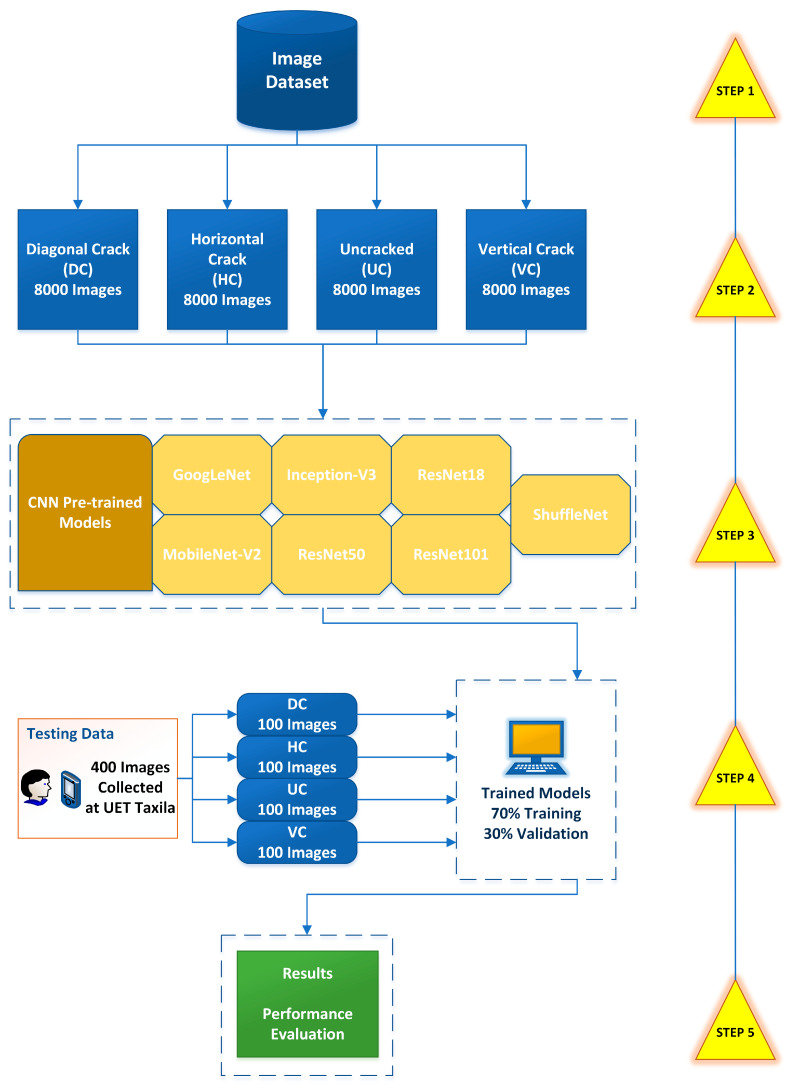
Proposed methodology.

Figure 1 outlines the five-step method utilized in this study. First, a dataset was obtained from the open-source SDNET2018 [57]. Next, the images were divided into four different groupings, diagonal crack (DC), horizontal crack (HC), uncracked (UC), and vertical crack (VC). Then, the selected pre-trained CNN models were acquired, trained, and validated on the dataset. Next, these models were validated on a crack dataset collected from the UET Taxila. The final step was to compute the confusion matrix of each model and measure its performance.

### 3.2. Dataset Acquisition

**Figure 2:**
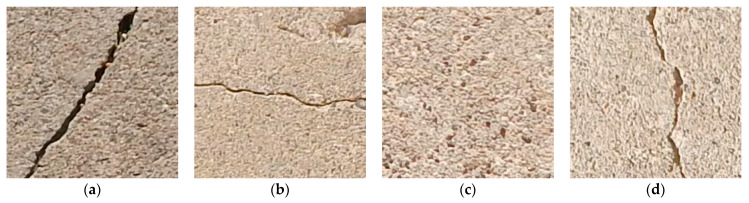
(**a**) Diagonal crack, (**b**) horizontal crack, (**c**) uncracked, (**d**) vertical crack.

In this study, the dataset was divided into three categories: training, validation, and testing. A 32,000-image dataset with 227 × 227 × 3 resolution was gathered from SDNET2018 [60], and another dataset of 400 images was collected from the UET Taxila. The 32,000-image dataset was divided into the DC, HC, UC, and VC categories depending on the orientation of cracks, as shown in Figure 2. Each category contained a dataset of 8000 images with equal resolution. The dataset containing 400 cracked and uncracked images collected from the UET Taxila was also categorized into four categories depending on the orientation of cracks. For testing, the 400-image dataset was divided into the DC, HC, UC, and VC categories equally depending on the orientation of cracks.

### 3.3. Training, Validation, and Testing Dataset

For CNN’s pre-trained models, 70% of the images in each category were selected randomly from the dataset, while the remaining 30% were applied for validation. The ratio of training and validation, 70–30%, was maintained for all the pre-trained models with the same epoch value of 3. However, for testing, 400 images were employed. The training option optimizer used was stochastic gradient descent with momentum, minimum batch size = 10, maximum number of epochs = 3, initial learning rate = 0.003, and the training data shuffled after every epoch.

### 3.4. Pre-Trained Models

**Figure 3:**
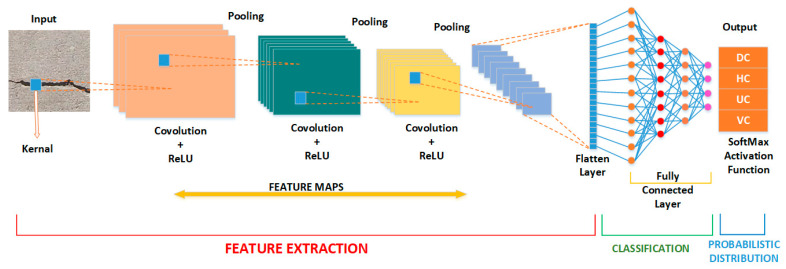
CNN architecture model.

CNN models have many fixed layers and convolutional blocks, consisting of convolutions, batch normalization, activation, ReLU, pooling, max pooling, average pooling, fully connected, soft-max layers, etc., as illustrated in Figure 3 [29]. While many pre-trained CNN models are available, only the most widely regarded models, including ResNet18, ResNet50, ResNet101, MobileNet-V2, GoogLeNet, Inception-V3, and ShuffleNet, were used in this study. Intending to improve performance on mobile devices, MobileNet-V2 is a CNN architecture. This design relies on a backward residual architecture, in which the bottleneck levels are the links between the residual layers. A source of non-linearity is filtered out in the intermediate expansion layer by utilizing lightweight depth-wise convolutions applied to features. MobileNet-V2’s overall structure consists of a 32-filter fully convolutional first layer and then 19 layers of residual bottlenecks. Inception-V3 is from the Inception family that uses Label Smoothing, factorized 7 × 7 convolutions, and an auxiliary classifier to transport label information further down the network. ResNet models introduce the idea of residual learning [61]. ResNets are trained to learn residual functions by referencing the inputs to each layer in the network. Residual nets allow stacked layers to match a residual mapping rather than assuming each layer directly fits a desired underlying mapping. To create a network, they pile residual blocks one on top of another; for instance, a ResNet50 has fifty layers. GoogLeNet is based on the Inception design. It employs Inception modules, which let the network choose from various convolutional filter sizes in each block. An Inception network stacks these modules on top of one other, with max-pooling layers with stride two to occasionally reduce the grid’s resolution. ShuffleNet was developed specifically for use on mobile devices with minimal processing capacity. The design applies two procedures, pointwise group convolution and channel shuffle, to improve efficiency without sacrificing accuracy. All the pre-trained models have different specifications, as summarized in Table 2. There are several layers in ResNet’s architecture, which are indicated by the name. MobileNet-V2 is a CNN model with a minimal level of complexity designed with on-device or embedded applications and limited resources to consider [61]. More than one million images from the ImageNet dataset served as the training data for these pre-trained networks. The trained networks can categorize images into 1000 different categories, including numerous different animals, a keyboard, a mouse, and a pencil. These networks have therefore acquired extensive properties that accurately classify a variety of images.

## 4. Results and Discussion

In this study, seven different pre-trained CNN models, including GoogLeNet, Inception-V3, MobileNet-V2, ResNet18, ResNet50, ResNet101, and ShuffleNet, were trained, validated, and tested on a comprehensive dataset of images to detect and classify cracks. The same dataset of images and computer specifications was used to evaluate these pre-trained models with the same ratio of image division. The performance of each CNN for detecting cracks and orientation was measured by computing the confusion matrix, accuracy, precision, recall, and F1 score, and recording the computational time during the training of each model with constant values for the number of epochs and other parameters. The networks employed in this investigation were pre-trained using ImageNet data and were taken from the MathWorks website [62]. All tests were run on a desk-top workstation with an Intel Core i3 9th generation processor, 16 GB of RAM, and a Nvidia GTX 1650 super 4 GB graphics card using MATLAB 2020.

Accuracy, precision, specificity, recall, and F1 score were the four statistical parameters used to evaluate the four-class classification performance of pre-trained CNNs. Model accuracy is a performance parameter for ML classification models that is defined as the proportion of true positives and true negatives to the total number of positive and negative observations. In other words, accuracy indicates how often we may anticipate that our model would accurately predict an event, relative to the total number of times it has made predictions. Accuracy [40] is the rate of all the actual predicted values to the total number of predictions done and is calculated as:

(1) $$Accuracy=\frac{TP+TN}{TP+TN+FP+FN}$$

where *TP* is truly positive, *TN* is a true-negative, *FP* is a false-positive, and *FN* is a false-negative prediction value.

Accuracy of GoogLeNet for predicting the DC, HC, UC, and VC was 92%, 93%, 88%, and 92%, respectively. Similarly, the accuracy of MobileNet-V2 for predicting the DC, HC, UC, and VC was 86%, 91%, 87%, and 84%, respectively; for Inception-V3 was 96%, 94%, 92%, and 96%, respectively; for ShuffleNet was 82%, 91%, 90%, and 96%, respectively; for ResNet50 was 88%, 97%, 92%, and 86%, respectively; for ResNet101 was 95%, 95%, 92%, and 94%, respectively; and for ResNet18 was 84%, 90%, 85%, and 89%, respectively. These accuracies are compared in Table 3. These results demonstrated that Inception-V3 and ResNet101 performed better than other models with accuracies greater than 90%. Further pinpointing the best model, Inception-V3 performed well compared with ResNet101, with accuracies for detecting DC and VC greater for Inception-V3. Accuracy for detecting UC for both models remained the same. In contrast, accuracy for detecting HC for ResNet101 was 1% greater than Inception-V3.

The model precision score quantifies the fraction of accurately predicted positive labels. Precision is also referred to as the predictive value of the positive. Precision [40] is calculated as:

(2) $$Precision=\frac{TP}{TP+FP}$$

Precision of MobileNet-V2 for predicting the DC, HC, UC, and VC was 92%, 88%, 47%, and 67%, respectively; for Inception-V3 was 98%, 96%, 67%, and 93%, respectively; for GoogLeNet was 94%, 89%, 53%, and 95%, respectively; for ShuffleNet was 97%, 65%, 60%, and 95%, respectively; for ResNet50 was 98%, 91%, 69%, and 68%, respectively; for ResNet101 was 86%, 99%, 71%, and 95%, respectively; and for ResNet18 was 98%, 74%, 40%, and 85%, respectively. These precisions are compared in Table 3. An interesting fact can be observed in Table 3. The precision value for detecting UC images was the lowest for each model compared with the other three classes. The lowest precision value was 40% for ResNet18 to detect UC images and the highest was for ResNet101.

The model’s ability to properly predict positives out of actual positives was measured by the model recall score. This differs from precision, which counts the proportion of accurate positive predictions among all positive predictions given by models. Recall [40] is calculated as:

(3) $$Recall=\frac{TP}{TP+FN}$$

Recall of MobileNet-V2 for predicting the DC, HC, UC, and VC was 65%, 77%, 100%, and 68%, respectively; for Inception-V3 was 88%, 81%, 100%, and 89%, respectively; for GoogLeNet was 79%, 84%, 100%, and 78%, respectively; for ShuffleNet was 58%, 97%, 100%, and 90%, respectively; for ResNet50 was 67%, 97%, 100%, and 75%, respectively; for ResNet101 was 93%, 83%, 96%, and 83%, respectively; and for ResNet18 was 62%, 85%, 100%, and 75%, respectively. These recalls are compared in Table 3. Recall value for the detection of UC class was 100% for all the models except ResNet101 for which its value was 96%.

F1 score represents the model score as a function of the recall and precision scores. F1 score is a performance metric for models that provides equal weight to precision and recall when evaluating its accuracy. F1 score [40] is the harmonic mean of precision and recall, and is calculated as:

(4) $$F1\;score=2\left(\frac{Recall\times Precision}{Recall+Precision}\right)$$

F1 score of MobileNet-V2 for predicting the DC, HC, UC, and VC was 76%, 82%, 64%, and 68%, respectively; for Inception-V3 was 93%, 88%, 80%, and 91%, respectively; for GoogLeNet was 86%, 86%, 69%, and 86%, respectively; for ShuffleNet was 73%, 78%, 75%, and 92%, respectively; for ResNet50 was 80%, 94%, 82%, and 71%, respectively; for ResNet101 was 90%, 90%, 82%, and 88%, respectively; and for ResNet18 was 76%, 79%, 57%, and 79%, respectively. Table 3 provides these F1 scores for DC, HC, UC, and VC for all the studied CNN architectures. Both Inception-V3 and ResNet101 had a greater value of F1 score compared with others. For DC and VC classes, Inception-V3’s F1 score was greater than ResNet101, while HC and UC’s F1 score value was greater for the latter.

**Figure 4:**
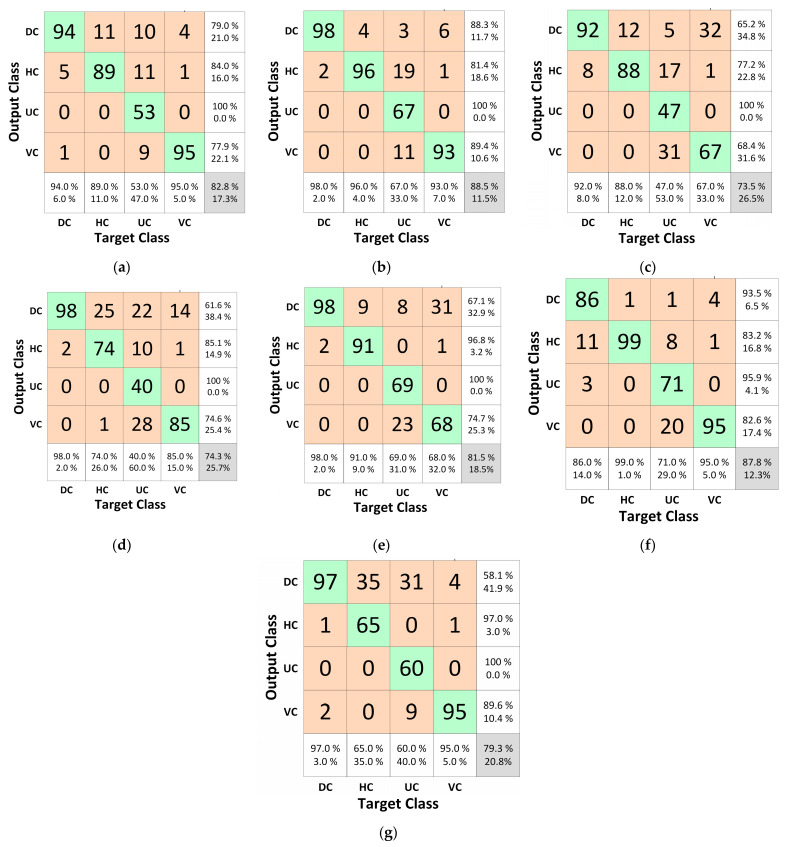
Confusion matrix plots for selected architecture: (**a**) GooLeNet, (**b**) Inception-V3, (**c**) MobileNet-V2 (**d**) ResNet18, (**e**) ResNet50, (**f**) ResNet101, (**g**) ShuffleNet.

The confusion matrix plots based on testing images are indicated in Figure 4. The overall accuracies of Inception-V3, MobileNet-V2, GoogLeNet, ResNet18, ResNet50, ResNet101, and ShuffleNet were 88.5%, 73.5%, 82.8%, 74.3%, 81.5%, 87.8%, and 79.3%, respectively. These results indicate that Inception-V3 had the highest accuracy among all the models, while MobileNet-V2 had the lowest overall accuracy value. From the confusion matrix for all the models, it was observed that models did not perform well in detecting class UC. The best model for the right classification of the DC class was ResNet101, which rightly identified 71 UC images out of 100.

**Figure 5:**
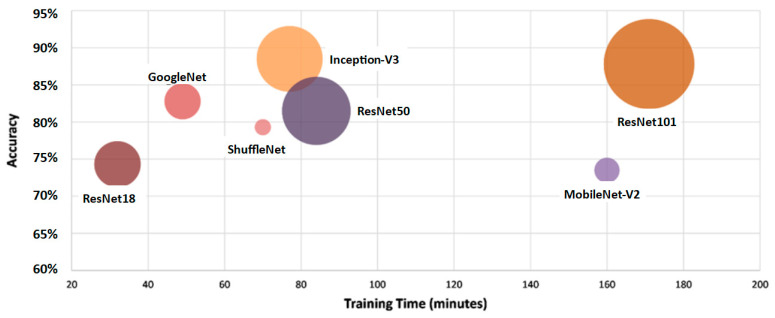
Accuracy vs. training time and size of model.

The plot between the CNN model’s accuracy, time consumed on training, and model size is reported in Figure 5. The training time and size of the model of ResNet101 was the highest among all the other models under study, equal to 171 min for training and size of 167 MB. ResNet18 took a minimum time to train, equal to 32 min. Inception-V3 provided the best result regarding the accuracy, model size, and time taken for training. As its training time was lesser than the model with the highest training time, its accuracy rate was the highest among all, and its size was also smaller than the one with a larger size.

**Figure 6:**
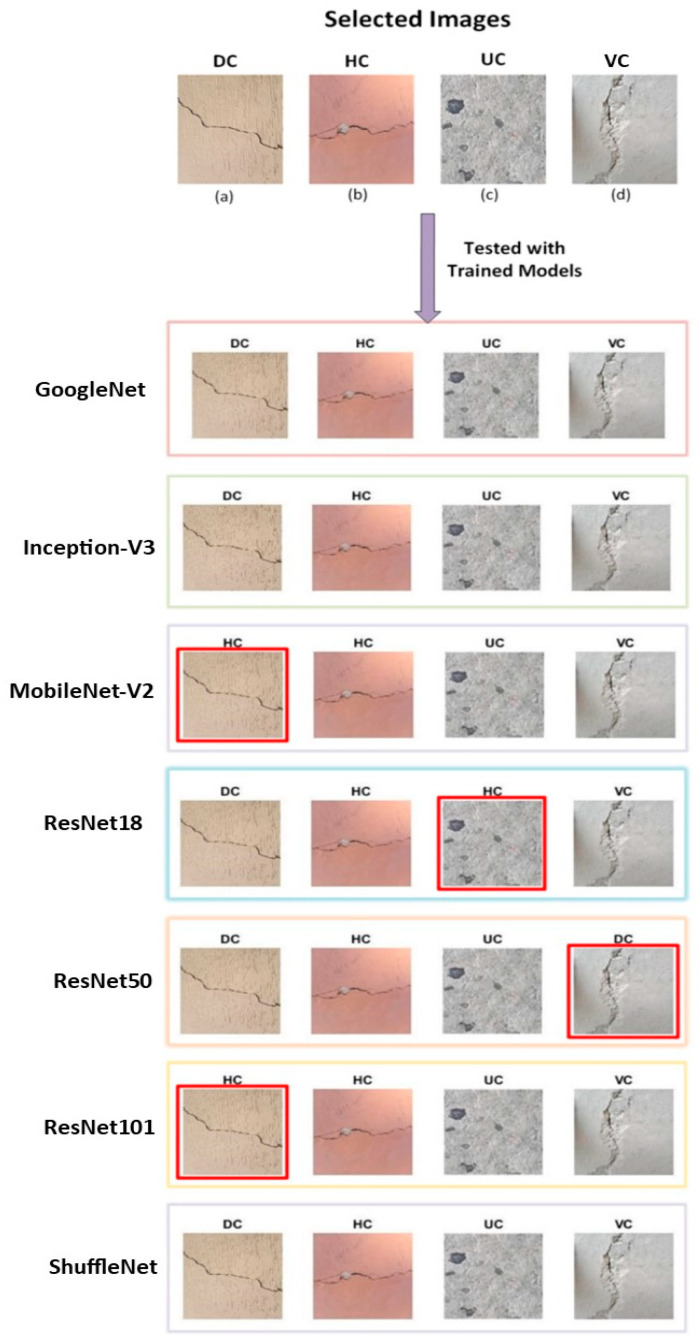
Selected images and results after passing through trained models, while red boxes show poor predictions against each model.

One image was randomly taken from each class from the testing dataset and tested on the trained models. The results are presented in Figure 6. The figure elaborates that Inception-V3, GoogLeNet, and ShuffleNet rightly identified the image according to the class to which it belongs. MobileNet-V2 and ResNet101 were confused by the DC class and identified as HC. ResNet18 detected the UC class image as HC, while ResNet50 confused the VC image and detected it as DC. All the models rightly identified the HC image. None of the models confused the HC image with the VC image and vice versa.

## 5. Conclusions

This research investigated the classification of cracks using pre-trained CNN models. The performance of seven pre-trained models, including GoogLeNet, MobileNet-V2, Inception-V3, ResNet18, ResNet50, ResNet101, and ShuffleNet were evaluated using the same dataset, computer specifications, and other parameters. After comparing the performance of CNN architectures for the classification of the crack images based on accuracy, precision, recall, F1 score, size of models, and training time, the best model was suggested.

The Inception-V3 outperformed the analyzed CNN models with accuracies of 96%, 94%, 92%, and 96% for the DC, HC, UC, and VC classifications, respectively. From the confusion matrix, it had the best overall performance of 88.5%.

From a practical point of view, it is important to detect whether the concrete surface is cracked. Then, autonomously identifying the orientation of the crack could help predict the cause of failure, i.e., flexural stress, shear stress, or combined.

## Figures and Tables

**Table 1:** Literature review.

Method	Domain	Dataset	Image Features	Results	Reference
FFT, FHT, Sobel, and Canny edge detector	Concrete Bridge	50	640 × 480	FHT more reliable Accuracy = 86%	[26]
SVM	Bridge deck	118	-	Accuracy = 76%	[27]
VGG16	Street Images	48,000	200 × 200	Accuracy = 98.6%	[28]
Fully Convolutional Network VGG16, Inception-V3, and ResNet	Concrete Cracks	40,000	227 × 227	VGG16 Accuracy = 99.9%	[29]
Canny and Sobel Edge detection	Concrete Cracks	40,000	256 × 256	Accuracy = 98.2%	[30]
CNN and SVM	Masonry Structure	6002	96 × 96	Accuracy = 86%	[31]
AlexNet and ChaNet	Concrete Cracks	125	256 × 256	ChaNet Accuracy = 7.91%	[33]
Deep CNN with eight layers Convolution, Pooling, Relu, and SoftMax	Concrete Cracks	40,000	256 × 256 × 3	Accuracy = 98%	[34]
VGG16	Pavement Cracks	1056	3072 × 2048	Accuracy = 87%	[35]
ResNet18, ResNet50, ResNet101, and MobileNet-V2	Concrete and Pavement Cracks	32,000	256 × 256 × 3	ResNet50 Accuracy = 86.2%	[36]
ResNet50	Pavement Cracks	48,000	256 × 256 × 3	Accuracy = 99.8%	[37]
AlexNet, VGG16, VGG19, GoogLeNet, ResNet50, ResNet101, and ResNet152	Masonry walls and Concrete Floors	40,000	224 × 224	VGG16 Average Accuracy = 96%	[39]
SVM and MDNMS	Road Cracks	7250	4000 × 1000	Precision = 98.29%	[56]
GoogLeNet, CNN, and FPN	Concrete Cracks	128,000	6000 × 4000	Precision = 80.13%	[57]
STRUM, SVM, AdaBoost, and Random Forest	Concrete Bridge	100	1920 × 1280	Accuracy = 95%	[58]
SVM and CNN	Pavement Cracks	500	3264 × 2448	Accuracy = 91.3%	[59]
GoogLeNet, MobileNet-V2, and Inception-V3	Concrete and Pavement Cracks	48,000	256 × 256 × 3	Inception-V3 Accuracy = 97.2%	[41]

**Table 2:** Specifications of pre-trained models.

Network	Image Input Size	Parameters (Millions)	Size (MB)	Depth (Layers)
GoogLeNet	224 × 224	7.0	27	22
Inception-V3	299 × 299	23.9	89	48
MobileNet-V2	224 × 224	3.5	13	53
ResNet18	224 × 224	11.7	44	18
ResNet50	224 × 224	25.6	96	50
ResNet101	224 × 224	44.6	167	101
ShuffleNet	224 × 224	1.4	1.4	50

**Table 3:** Results for accuracy, precision, recall, and F1 score.

Sr. No.	CNN Architecture	Class	Accuracy	Precision	Recall	F1 Score
**1**	**GoogLeNet**	DC	92%	94%	79%	86%
		HC	93%	89%	84%	86%
		UC	88%	53%	**100%**	69%
		VC	92%	**95%**	78%	86%
**2**	**MobileNet-V2**	DC	86%	92%	65%	76%
		HC	91%	88%	77%	82%
		UC	87%	47%	**100%**	64%
		VC	84%	67%	68%	68%
**3**	**Inception-V3**	DC	**96%**	**98%**	88%	**93%**
		HC	94%	96%	81%	88%
		UC	**92%**	67%	**100%**	80%
		VC	**96%**	93%	89%	91%
**4**	**ResNet18**	DC	84%	**98%**	62%	76%
		HC	90%	74%	85%	79%
		UC	85%	40%	**100%**	57%
		VC	89%	85%	75%	79%
**5**	**ResNet50**	DC	88%	**98%**	67%	80%
		HC	**97%**	91%	**97%**	**94%**
		UC	**92%**	69%	**100%**	**82%**
		VC	86%	68%	75%	71%
**6**	**ResNet101**	DC	95%	86%	**93%**	90%
		HC	95%	**99%**	83%	90%
		UC	**92%**	**71%**	96%	**82%**
		VC	94%	**95%**	83%	88%
**7**	**ShuffleNet**	DC	82%	97%	58%	73%
		HC	91%	65%	**97%**	78%
		UC	90%	60%	**100%**	75%
		VC	**96%**	**95%**	**90%**	**92%**

## Data Availability

Not applicable.
